# Simple Point-of-Care Nucleic Acid Amplification Test for Rapid SARS-CoV-2 Infection Diagnosis

**DOI:** 10.3390/diagnostics13183001

**Published:** 2023-09-20

**Authors:** Hyunseul Jee, Minkyeong Choi, In Su Park, Junmin Lee, Woong Sik Jang, Chae Seung Lim

**Affiliations:** 1 BK21 Graduate Program, Department of Biomedical Sciences, College of Medicine, Korea University, Seoul 02841, Republic of Korea; jhs603@korea.ac.kr (H.J.); min508k@naver.com (M.C.); in2269@naver.com (I.S.P.); dlwnsals15@korea.ac.kr (J.L.); 2 Emergency Medicine, College of Medicine, Korea University Guro Hospital, Seoul 08308, Republic of Korea; 3 Department of Laboratory Medicine, College of Medicine, Korea University Guro Hospital, Seoul 08308, Republic of Korea

**Keywords:** point-of-care, SARS-CoV-2, loop-mediated isothermal amplification, nucleic acid lateral flow

## Abstract

After three years of the SARS-CoV-2 pandemic, the demand for developing field-deployable point-of-care (PoC) molecular diagnostic tests has increased. Although RT-qPCR is the molecular diagnostic gold standard and is accurate, it is not readily applied to point-of-care testing (POCT). Meanwhile, rapid diagnostic kits have the disadvantage of low sensitivity. Recently, rapid isothermal nucleic acid amplification technology has emerged as an alternative for rapid diagnosis. Here, we developed a rapid SARS-CoV-2 reverse transcription loop-mediated isothermal amplification (RT-LAMP)-lateral flow assay (LFA) kit. This kit includes a Chelex-100/boiling nucleic acid extraction device and a one-step amplification detection apparatus capable of performing the entire process, from RNA extraction to detection, and diagnosing SARS-CoV-2 infection within 40 min without contamination. The detection limits of the rapid SARS-CoV-2 RT-LAMP-LFA kit were 100 plaque-forming units (PFUs) mL^−1^ and 10^−1^ PFU mL^−1^ for RNA samples extracted using the Chelex-100/boiling nucleic acid extraction device and commercial AdvansureTM E3 system, respectively. The sensitivity and specificity of the rapid SARS-CoV-2 RT-LAMP-LFA kit were 97.8% and 100%, respectively. Our SARS-CoV-2 RT-LAMP-LFA kit exhibited high sensitivity and specificity within 40 min without requiring laboratory instruments, suggesting that the kit could be used as a rapid POC molecular diagnostic test for SARS-CoV-2.

## 1. Introduction

Severe acute respiratory syndrome coronavirus 2 (SARS-CoV-2) emerged in late 2019. It is a positive-sense, single-stranded RNA virus [1]. After three years of the SARS-CoV-2 pandemic, the demand for developing field-deployable point-of-care (PoC) molecular diagnostic tests has increased to improve early detection, quarantine, treatment, and effective control of infectious disease transmission [2,3]. RT-qPCR, the molecular diagnostic gold standard, is accurate but difficult to apply to point-of-care testing (POCT) due to the requirement for expensive equipment, skilled labor, and a long turnaround time [4]. In contrast, rapid diagnostic kits (using an antibody) are rapid but have the disadvantage of low sensitivity [5]. Therefore, rapid isothermal nucleic acid amplification technology (INAAT), similar in terms of sensitivity to RT-qPCR, can be rapidly used diagnostically and has emerged as an alternative [6,7,8].

Unlike conventional PCR, INAAT technology can amplify a target at a constant temperature, making it suitable for on-site diagnosis. INAAT encompasses various methods, such as strand displacement amplification (SDA) [9], helicase-dependent amplification (HDA) [10], recombinase polymerase amplification (RPA) [11], and loop-mediated amplification (LAMP) [12]. Among these, the LAMP method has been widely applied for diagnosing infectious diseases because of its relatively higher sensitivity and specificity than other INAAT methods [13,14]. Many studies have been conducted to increase the sensitivity and specificity of LAMP using various biosensors, such as fluorescent probes, colorimetric indicators, and lateral flow assays (LFA) [15,16,17,18].

Previous studies for developing POC INAAT assays have primarily been conducted in central laboratories by personnel skilled in molecular testing and sensitive to the detection of amplicon/sample contamination [19,20,21]. However, since actual POC tests are typically performed by healthcare providers with limited experience in molecular testing and in general spaces rather than central laboratories, they are prone to external contamination or may require the use of laboratory equipment that is difficult to use. Therefore, isothermal amplification molecular diagnostic kits for field diagnosis should require minimal external exposure from sample nucleic acid extraction to detection and should be easy to use by end medical service providers with limited experience and minimal equipment. Recently, a platform technology that can rapidly detect molecules from patient samples in the field using LAMP-LFA technology with new nucleic acid extraction techniques has been studied [22,23]. However, these studies have not excluded the possibility of contamination by LAMP amplicons from tubes being opened during the detection process.

In this study, we developed a rapid SARS-CoV-2 RT-LAMP-LFA kit, including a Chelex-100/boiling nucleic acid extraction device and a one-step amplification-detection device that can diagnose SARS-CoV-2 within 40 min from RNA extraction to detection. This kit has extremely limited external exposure throughout the process to prevent contamination. Of all the steps from nucleic acid extraction to detection, only one filtering step involves opening the tube lid; in this case, the filter tube is connected simultaneously as the lid is opened. The detection limit of the rapid SARS-CoV-2 RT-LAMP-LFA kit was confirmed using clinical nasopharyngeal (NP) samples spiked with serially diluted SARS-CoV-2. The sensitivity and specificity of the rapid SARS-CoV-2 RT-LAMP-LFA kit were compared with those of a SARS-CoV-2 RT-LAMP assay and the commercial Allplex^TM^ SARS-CoV-2 assay for SARS-CoV-2 clinical NP samples.

## 2. Materials and Methods

### 2.1. SARS-CoV-2 and Clinical Samples

A total of 20 SARS-CoV-2 strains, including one wild-type and 19 mutants, were obtained from the Korea Disease Control and Prevention Agency (KDCA) (Table S1). For clinical testing, we used clinical SARS-CoV-2 NP samples (*n* = 92) and clinically normal NP samples (negative control group, *n* = 100) collected from SARS-CoV-2-infected (from June 2022 to July 2022) and non-infected (from February 2018 to November 2019) patients at Korea University Guro Hospital. All clinical samples were confirmed using the Allplex^TM^ SARS-CoV-2 assay (Seegene Inc., Seoul, Republic of Korea). For cross-reactivity testing, a total of 42 clinical respiratory samples (9 of coronavirus (229E, NL63, and OC43), 3 of influenza virus A/H1N1, 3 of influenza virus A/H3N2, 3 of influenza virus B, 3 of respiratory syncytial virus (RSV) A, 3 of RSV B, 3 of adenovirus (AdV), 3 of parainfluenza virus (PIV), 3 of human bocavirus (HboV), 3 of human enterovirus (HEV), 3 of human rhinovirus (HRV), and 3 of metapneumovirus (MPV)) were collected from Korea University Guro Hospital and confirmed by PCR using the Anyplex^TM^ II RV16 detection kit (Seegene Inc., Seoul, Republic of Korea). This study was conducted in accordance with the guidelines of the Declaration of Helsinki and was approved by the Institutional Review Board of Korea University Guro Hospital (approval number: 2019GR0055).

### 2.2. RNA Extraction

**Figure 1:**
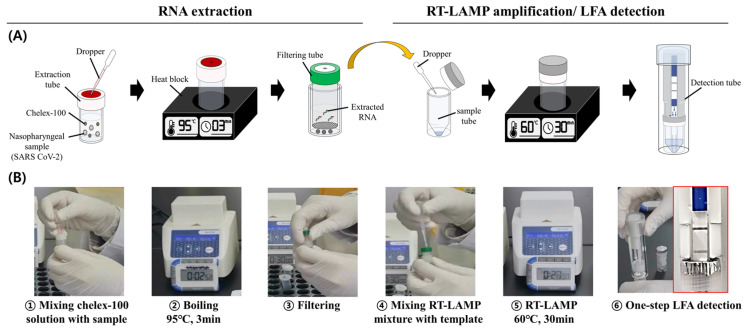
Process of the SARS-CoV-2 RT-LAMP-LFA kit. (**A**) Schematic diagram of the process. (**B**) Captured images from the video taken during the experimental process (Video S1).

RNA extraction was performed using two different methods: the Advansure^TM^ E3 system (LG chemistry, Seoul, Republic of Korea) and the Chelex-100/boiling nucleic acid extraction device, which was a slightly modified version of a previously reported device [24]. First, RNA was extracted from the samples using the Advansure^TM^ E3 system according to the manufacturer’s instructions, and RNA was eluted in 100 μL of elution buffer. Second, RNA was extracted from the samples using a Chelex-100/boiling nucleic acid extraction device (Figure 1A,B). The Chelex-100 Resin (75–150 µm, Bio-Rad Laboratories, Hercules, CA, USA) effectively inhibits nuclease activity in complex samples and stabilizes samples for downstream PCR applications by binding divalent metal ions, such as Mg^2+^, that are required for metallo-nuclease activity. Briefly, 200 µL of clinical or virus-spiked NP samples were added to a tube containing 200 µL of 20% Chelex-100 Resin solution (10 mM Tris-HCl, 1 mM EDTA, pH 8.0), and the tube was tapped five times. Tubes containing sample mixtures were heated for 3 min at 95 °C in a heating block and then tapped five times. After filtration through a 3 µm polycarbonate track-etched membrane filter (Whatman, Haverhill, MA, USA), the supernatant was transferred to a new tube for subsequent experiments. The filtration step was performed using a SEPARA^®^ tube (GVS, Bologna, Italy) modified by attaching a 3 µm polycarbonate track-etched membrane to the filtering unit using instant adhesive (UNITECH, Ansan, Gyeonggi, Republic of Korea) after removing the existing filter (0.2 µm).

### 2.3. Design of a One-Step Amplification-Detection Device for the Rapid SARS-CoV-2 RT-LAMP-LFA Kit

**Figure 2:**
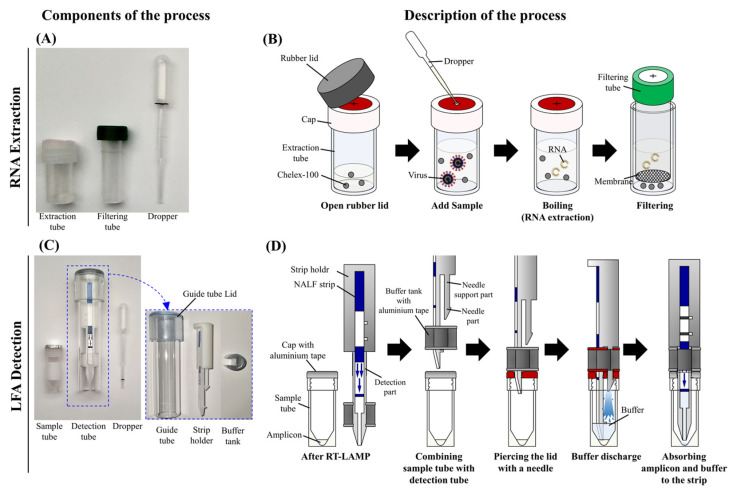
Components and illustration of the SARS-CoV-2 RT-LAMP-LFA kit. Components of the Chelex-100/boiling nucleic acid extraction device (**A**) and operational steps (**B**). Components of the one-step amplification-detection device (**C**) and operational steps (**D**).

The one-step amplification-detection device consisted of a sample tube and a detection tube (Figure 2C). The sample tube contained an external threaded cap with a hole in the cap, which was sealed with aluminum tape to prevent evaporation of the sample during the LAMP process; it was designed to be easily torn by the needle of the strip holder. The detection tube comprised a lid, guide tube, strip holder, PCRD flex nucleic acid test strip (Abingdon Health, York, UK), and a buffer tank. The buffer tank was sealed with aluminum tape at the top and bottom after the buffer was injected. The strip holder, which was attached to the lid of the guide tube, included a detection component that could be used to attach the strip and tear the aluminum tape on the cap of the sample tube and a needle that could be used to tear the aluminum tape on the lid of the buffer tank and the cap of the sample tube (Figure 2D). The PCRD flex strip, which detects carboxyfluorescein (FAM) and digoxigenin (DIG) signals, was attached to the strip holder using double-sided tape. The maximum diameter of the needle was smaller than that of the buffer tank, and the needle support was designed to be smaller than the maximum diameter of the needle so that the solution in the buffer tank flowed into the sample tube easily. The strip holder and buffer tank of the detection tube were designed using Sketchup (Trimble, Westminster, CO, USA) and fabricated using a Kings 450 Pro 3D printer (3D Solution, Yongin, Gyeonggi, Republic of Korea).

### 2.4. Real-Time RT-qPCR and RT-LAMP

In all tests, the performance of the rapid SARS-CoV-2 RT-LAMP-LFA kit was compared to that of the Allplex^TM^ SARS-CoV-2 assay and SARS-CoV-2 RT-LAMP assay using the CFX96 Touch Real-Time PCR detection system with a c1000 Touch Thermal Cycler (Bio-Rad Laboratories, Hercules, CA, USA). The Allplex^TM^ SARS-CoV-2 assay was performed according to the manufacturer’s instructions. The thermal cycling conditions of the Allplex^TM^ SARS-CoV-2 assay were as follows: reverse transcription at 50 °C for 20 min, inactivation at 95 °C for 15 min, 45 cycles of denaturation at 95 °C for 10 s, and annealing with fluorescence detection at 60 °C for 15 s and 72 °C for 10 s. The CoV-2 RT-LAMP assay and rapid SARS-CoV-2 RT-LAMP-LFA kit used the ELPIS RT-LAMP 2× Master Mix (Elpis-biotech, Daejeon, Republic of Korea). For the SARS-CoV-2 RT-LAMP, we used a previously designed LAMP primer set for the RdRP gene (Table S2) [25]. For the CoV-2 RT-LAMP assay, the reaction mixture was prepared with 15 μL of 2× master mix, 1.5 μL of SARS-CoV-2 RdRP gene LAMP primer mix A, and 5 μL of sample RNA (final reaction volume 30 μL). For the rapid SARS-CoV-2 RT-LAMP-LFA kit, the reaction mixture was prepared with 15 μL of 2× master mix, 1.5 μL of SARS-CoV-2 RdRP gene LAMP primer mix B, and 5 μL of sample RNA (final reaction volume: 30 μL). The composition of the SARS-CoV-2 RdRP gene LAMP primer mix A and B is indicated in Table S2. The SARS-CoV-2 RT-LAMP assay and rapid SARS-CoV-2 RT-LAMP-LFA were run on a CFX 96 Touch Real-Time PCR Detection System and a heating block (Beijing HiYi Technology, Beijing, China) at 60 °C for 30 min, respectively.

### 2.5. Limit of Detection (LOD) Tests

For LOD tests, SARS-CoV-2 (NCCP 43346, wild-type) was spiked into normal clinical NP samples (10^3^ PFU mL^−1^) and serially diluted 10-fold with clinical NP samples from 10^3^ PFU mL^−1^ to 10^−3^ PFU mL^−1^. SARS-CoV-2 RNA was extracted from the samples using two different methods: the Advansure^TM^ E3 system (Seegene Technologies, Walnut Creek, CA, USA) and Chelex-100/boiling nucleic acid extraction devices. The LOD of the rapid SARS-CoV-2 RT-LAMP-LFA kit was compared with those of the Allplex^TM^ SARS-CoV-2 and SARS-CoV-2 LAMP assays for two types of SARS-CoV-2 RNA extracted with the Advansure^TM^ E3 system and Chelex-100/boiling nucleic acid extraction devices. All tests were repeated three times and determined as the minimum concentration in a 10-fold dilution series at which three replicates were amplified.

## 3. Results

### 3.1. Rapid SARS-CoV-2 RT-LAMP-LFA Kit

The rapid SARS-CoV-2 RT-LAMP-LFA kit involves RNA extraction using the Chelex-100/boiling nucleic acid extraction device and LAMP amplification/detection using the one-step amplification-detection device (Figure 1). Clinical samples (200 µL) were added dropwise to a tube containing 200 µL of 20% Chelex-100. After heating the tube for 3 min at 95 °C, the mixture was filtered using the filtering unit of the Chelex-100/boiling nucleic acid extraction device. After injecting 5 µL of the RNA sample dropwise into the sample tube containing LAMP reagents, the sample tube was incubated for 30 min at 60 °C. The detection tube was then plugged into the sample tube. Thereafter, the aluminum tape on the buffer tank and the sample tube lids were opened by the needle portion of the strip holder, and the buffer flowed out to the bottom of the sample tube. Finally, the LAMP product and buffer mixture were absorbed into the strip, and the result was analyzed (Figure 1, Video S1).

### 3.2. Performances of the Chelex-100/Boiling Nucleic Acid Extraction Device

**Figure 3:**
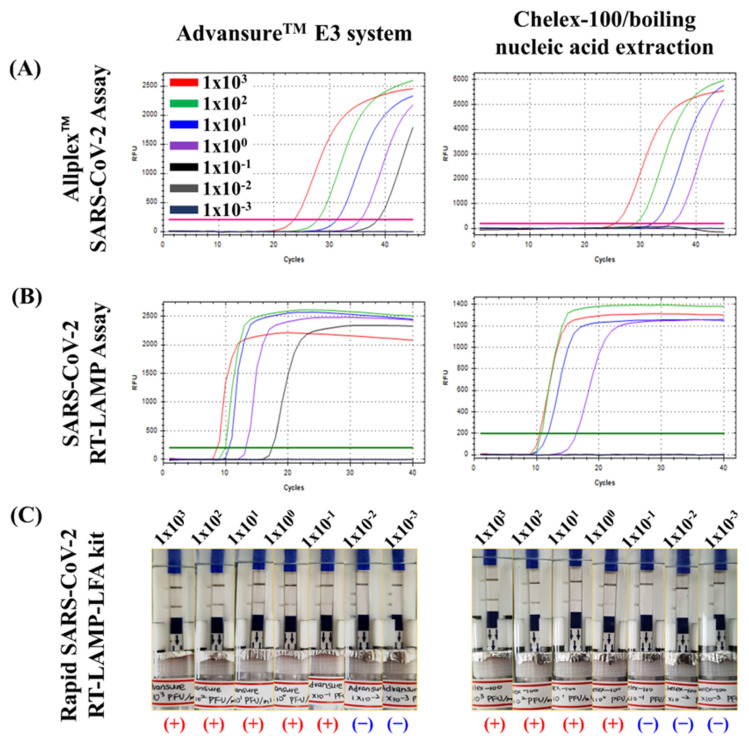
Comparison of the LOD of three kits for RNA samples extracted using the Chelex-100/boiling nucleic acid extraction device and the Advansure^TM^ E3 system. The LOD of the Allplex SARS-CoV-2 assay (**A**), SARS-CoV-2 RT-LAMP assay (**B**), and rapid SARS-CoV-2 RT-LAMP-LFA kit (**C**) were tested using RNA samples extracted with the Chelex-100/boiling nucleic acid extraction device and Advansure^TM^ E3 system, respectively.

Using the Allplex™ SARS-CoV-2 assay for quantitative analysis, the performance (%) of the Chelex-100/boiling nucleic acid extraction device was evaluated based on cycle threshold (Ct) values and predetermined concentrations (PFU mL^−1^) of serially diluted RNA samples extracted using the Advansure^TM^ E3 system (10^3^–10^−3^ PFU mL^−1^) (Figure 3A). The LOD of the Allplex^TM^ SARS-CoV-2 assay, the SARS-CoV-2 LAMP assay, and the rapid SARS-CoV-2 RT-LAMP-LFA kit were 100 PFU mL^−1^ and 10^−1^ PFU mL^−1^ against RNA samples extracted using the Chelex-100/boiling nucleic acid extraction device and Advansure^TM^ E3 systems, respectively (Figure 3, Table 1). In the LOD tests, the Chelex-100/boiling nucleic acid extraction device exhibited ten times poorer performance than the Advansure^TM^ E3 system in all three assays. Although the Chelex-100/boiling nucleic acid extraction device showed lower efficiency than the commercial Advansure^TM^ E3 system, the detection limit (10^0^ PFU mL^−1^) of the rapid SARS-CoV-2 RT-LAMP-LFA kit using RNA extracted by the Chelex-100/boiling nucleic acid extraction device was 100 times lower than the detection limit (10^2^ PFU mL^−1^) of the SARS-CoV-2 antigen rapid test kit currently used in the field (Biozentech Inc., Seoul, Republic of Korea) (Figure S1).

### 3.3. Clinical Performance of the Rapid SARS-CoV-2 RT-LAMP-LFA Kit

To confirm the clinical performance of the rapid SARS-CoV-2 RT-LAMP-LFA kit, the sensitivity and specificity of the system were compared to those of the SARS-CoV-2 RT-LAMP assay and Allplex™ SARS-CoV-2 assay using 192 clinical samples, including 92 NP swab samples from individuals with SARS-CoV-2 and 100 clinical NP swab samples from individuals without viral respiratory infections (Table 2, Figure S2). For sensitivity and specificity testing, RNA was extracted from NP clinical samples using the Chelex-100/boiling nucleic acid extraction device. For SARS-CoV-2 clinical samples (*n* = 92), the sensitivity of the Allplex™ SARS-CoV-2 assay for RdRP was 100%. The sensitivities of the SARS-CoV-2 RT-LAMP and rapid SARS-CoV-2 RT-LAMP-LFA kits were 97.8%. For the normal clinical samples (*n* = 100), the specificities of all three assays were 100%.

### 3.4. Cross-Reactivity Test

To confirm the absence of cross-reactivity with other common respiratory viruses, NP swabs from 42 patients with known infections of nine coronaviruses (229E, NL63, and OC43), nine influenza viruses (Inf A/H1N1, Inf A/H3N2, Inf B), six RSV A/B, three AdV, three PIV, three HBoV, three HEV, three HRV, and three MPV were tested using the rapid SARS-CoV-2 RT-LAMP-LFA kit, SARS-CoV-2 RT-LAMP assay, and Allplex™ SARS-CoV-2 assay (Table 3, Figure S3). In this test, RNA was extracted from 42 NP swab samples using the Advansure™ E3 system to accurately identify cross-reactivity using more purified viral RNA. All three molecular diagnostic tests showed no cross-reactivity with other infectious viruses. However, a limitation of this study is that cross-reactivity testing was not conducted for *Haemophilus influenzae* type B or *Klebsiella pneumoniae*, which are known to co-infect with SARS-CoV-2.

## 4. Discussion

Point-of-care testing (POCT) refers to diagnostic tests conducted directly at or near the patient’s location, reducing the time and costs associated with traditional laboratory-based testing and providing more effective patient care [26]. In the field of infectious disease screening, POCT provides a faster and more sensitive diagnosis than conventional pathogen detection methods [27]. Novel POC nucleic acid testing, which is a combination of molecular biology technology and POCT technology, not only integrates nucleic acid extraction, amplification, and detection in samples but also has the advantages of portability, easy operation, rapidity, and low cost while ensuring high specificity and sensitivity [28,29]. These POC nucleic acid testing developments could help establish an extensive POC nucleic acid testing infrastructure in major primary care facilities and regional hospitals that are more readily accessible to patients [30].

Considering the urgent need for POCT in various resource-constrained environments, such as emergency rooms, general wards, or sites, the application of molecular diagnostic systems to real-world field conditions requires rapid and successive nucleic acid extraction, amplification, and detection processes from patient samples without sophisticated laboratory facilities. In particular, extracting nucleic acids from patient samples using commercial nucleic acid extraction kits is complicated and time-consuming, and automated nucleic acid extraction equipment (for example, the Advance E3 system) is difficult to apply for on-site diagnosis. Therefore, in this study, we developed a new nucleic acid extraction device based on the Chelex-100/boiled nucleic acid extraction method [24], capable of extracting nucleic acids without laboratory equipment. The device comprises a graduated dropper, sample tube, and filter tube for direct and rapid extraction of nucleic acids from patient samples without the use of pipettes. The SARS-CoV-2 RT-LAMP-LFA kit exhibited comparable sensitivity and specificity to those of the commercial Allplex™ SARS-CoV-2 assay, which was used to test RNA samples extracted using the Advance E3 system. Furthermore, the RT-LAMP-LFA detection device was designed to detect results without external exposure to the amplified product. Therefore, the developed nucleic acid device should be considered useful for POC molecular diagnostic testing.

According to the WHO POC guide for the diagnosis or confirmatory testing of acute or subacute SARS-CoV-2 infection, a detection limit of 10^3^ copies mL^−1^, sensitivity/specificity of 95%/98%, and time to result within 45 min are considered acceptable goals [31]. The experimental results of this study almost met these criteria. The rapid SARS-CoV-2 RT-LAMP-LFA kit could be confirmed within 40 min from nucleic acid extraction to diagnosis and showed 97.8% sensitivity and 100% specificity for clinical NP samples. However, we used PFUs for the detection limit experiment; thus, the exact copy number of the detection limit was unknown. Nevertheless, considering the previously reported detection limit (4 × 10^3^ copies mL^−1^) of the Allplex™ SARS-CoV-2 assay [32], the detection limit of the rapid SARS-CoV-2 RT-LAMP-LFA kit was estimated to be approximately 4 × 10^4^ copies mL^−1^. Although the detection limit of the rapid SARS-CoV-2 RT-LAMP-LFA kit did not reach the acceptable limit (10^3^ copies mL^−1^) of the WHO POCT guide, these results were 75 times lower than the detection limit of commercial SARS-CoV-2 LFA tests (approximately 3 × 10^6^ copies mL^−1^) [33]. Indeed, we confirmed that the LOD of the rapid SARS-CoV-2 RT-LAMP-LFA kit was 100 times lower than that of the SARS-CoV-2 antigen rapid test kit (Figure S1). In addition, this kit detected the RdRP gene of 19 SARS-CoV-2 variants (Table S3, Figure S4), and heating block experiments confirmed that the temperature was maintained within an error of ±1 °C by assessing temperature changes inside the plate for 30 min at 1 min intervals (Figure S5).

## 5. Conclusions

Here, we developed a rapid SARS-CoV-2 RT-LAMP-LFA kit capable of diagnosing SARS-CoV-2 within 40 min from RNA extraction to detection. The rapid SARS-CoV-2 RT-LAMP-LFA kit exhibited high sensitivity (97.8%) and specificity (100%) without laboratory instruments, such as a pipette or centrifuge. These results suggest that the rapid SARS-CoV-2 RT-LAMP-LFA kit could be used as a POC molecular diagnostic test for SARS-CoV-2 infection.

## Figures and Tables

**Table 1:** LOD analysis of the Allplex™ SARS-CoV-2 assay, SARS-CoV-2 RT-LAMP assay, and rapid SARS-CoV-2 RT-LAMP-LFA kit for RNA samples extracted using the Chelex-100/boiling nucleic acid extraction device and Advansure^TM^ E3 system (Range 10^3^–10^−3^ PFU mL^−1^).

PFU mL^−1^	Advansure ^TM^ E3 System			Chlex-100/Boiling Nucleic Acid Extraction		
	Allplex ™ SARS-CoV-2 Assay	RT-LAMP Assay	RT-LAMP -LFA Kit	Allplex ™ SARS-CoV-2 Assay	RT-LAMP Assay	RT-LAMP -LFA Kit
	Ct ^1^ (SD ^4^)	Ct (SD)	Results	Ct (SD)	Ct (SD)	Results
1 × 10^3^	22.57 ± 0.11	8.73 ± 1.27	P ^2^	24.45 ± 0.40	11.29 ± 1.61	P
1 × 10^2^	27.03 ± 0.69	9.91 ± 1.25	P	28.22 ± 0.71	11.92 ± 1.66	P
1 × 10^1^	30.44 ± 0.29	10.85 ± 1.58	P	31.42 ± 0.52	13.50 ± 2.48	P
1 × 10^0^	34.54 ± 0.60	13.09 ± 1.73	P	35.03 ± 0.84	18.38 ± 5.62	P
1 × 10^−1^	37.84 ± 0.52	21.27 ± 2.70	P	N/A	N/A	N/A
1 × 10^−2^	N/A ^3^	N/A	N/A	N/A	N/A	N/A
1 × 10^−3^	N/A	N/A	N/A	N/A	N/A	N/A

^1^ Ct: Cycle threshold, ^2^ P: Positive results, ^3^ N/A: Not applicable, SD ^4^: Standard deviation.

**Table 2:** Comparison of the clinical performance of the Allplex™ SARS-CoV-2 assay, SARS-CoV-2 RT-LAMP assay, and rapid SARS-CoV-2 RT-LAMP-LFA kit for clinical samples.

Assay	Infected (*n* = 92)		Non-Infected (*n* = 100)		PPV **	NPV **
	P/N *	Sensitivity	P/N	Specificity		
Allplex™ SARS-CoV-2 assay	92/0	100%	0/100	100%	100%	100%
SARS-CoV-2 RT-LAMP assay	90/2	97.8%	0/100	100%	100%	93.7%
Rapid SARS-CoV-2 RT-LAMP-LFA kit	90/2	97.8%	0/100	100%	100%	93.7%

* “P” and “N” indicate a positive and negative reaction, respectively. ** “PPV” and “NPV” indicate a positive and negative predictive value of the reaction, respectively.

**Table 3:** Cross-reactivity of the rapid SARS-CoV-2 RT-LAMP-LFA kit, SARS-CoV-2 RT-LAMP assay, and Allplex™ SARS-CoV-2 assay for SARS-CoV-2 against other human infectious viruses.

Virus	Allplex™ SARS-CoV-2 Assay	SARS-CoV-2 RT-LAMP Assay	Rapid SARS-CoV-2 RT-LAMP-LFA Kit
	P/N *	P/N	P/N
CoV 229E	0/3	0/3	0/3
CoV NL63	0/3	0/3	0/3
CoV OC43	0/3	0/3	0/3
Inf A/H1N1	0/3	0/3	0/3
Inf A/H3N2	0/3	0/3	0/3
Inf B	0/3	0/3	0/3
RSV A	0/3	0/3	0/3
RSV B	0/3	0/3	0/3
PIV	0/3	0/3	0/3
AdV	0/3	0/3	0/3
HboV	0/3	0/3	0/3
HEV	0/3	0/3	0/3
HRV	0/3	0/3	0/3
MPV	0/3	0/3	0/3

* “P” and “N” indicate positive and negative reactions, respectively.

## Data Availability

The authors declare that all related data are available from the corresponding author upon reasonable request.

## Supplementary Materials

The following supporting information can be downloaded at: https://www.mdpi.com/article/10.3390/diagnostics13183001/s1. Table S1: SARS-CoV-2 wild-type and mutants obtained by the Korea Disease Control and Prevention Agency (KDCA); Table S2: SARS-CoV-2 RT-LAMP primers used in this study; Table S3: Results of the Allplex™ SARS-CoV-2 assay, SARS-CoV-2 RT-LAMP, and rapid SARS-CoV-2 RT-LAMP-LFA kit for 1 wild and 19 mutations of SARS-CoV-2; Figure S1: Limit of detection test of the rapid antigen kit for SARS-CoV-2 (NCCP 43346, wild-type) spiked in normal clinical nasopharyngeal samples (range: 10^3^–10^−3^ PFU ml^−1^). “+” and “−” indicate a positive and negative reaction, respectively; Figure S2: Clinical performance of the rapid SARS-CoV-2 RT-LAMP-LFA kit for clinical samples. (A) NP swab samples (*n* = 92) from individuals with SARS-CoV-2. (B) Clinical NP swab samples (*n* = 100) from individuals without viral respiratory infections. “+” and “−” indicate a positive and negative reaction, respectively; Figure S3: Cross-reactivity of the rapid SARS-CoV-2 RT-LAMP-LFA kit for SARS-CoV-2 with other human infectious viruses. “+” and “−” indicate a positive and negative reaction, respectively; Figure S4: Results of the rapid SARS-CoV-2 RT-LAMP-LFA kit for 1 wild and 19 mutations of SARS-CoV-2. “+” and “−” indicate a positive and negative reaction, respectively; Figure S5: Temperature changes in the heat block. The tests were repeated three times; Video S1: Experimental process of the SARS-CoV-2 RT-LAMP-LFA kit.
