# Supplementary material for: Simple Point-of-Care Nucleic Acid Amplification Test for Rapid SARS-CoV-2 Infection Diagnosis
Source: Diagnostics (Basel). 2023 Sep 20;13(18):3001. doi: 10.3390/diagnostics13183001 (PMC10529522; doi:10.3390/diagnostics13183001)
Supplement: Supplementary file 1 [file diagnostics-13-03001-s001.zip › Supplementary Materials.pdf]

Supplementary data

Table S1. SARS CoV-2 wild-type and mutants obtained by the Korea Disease Control and Prevention Agency (KDCA).

Pathogen	NCCP No.	Genotype (GSAID*)	Lineage
SARS-CoV-2	43346	GV (wild type)	B.1.177
SARS-CoV-2	43381	GRY	B.1.1.7
SARS-CoV-2	43382	GH	B.1.351
SARS-CoV-2	43383	GR	P.2
SARS-CoV-2	43384	GH	B.1.427
SARS-CoV-2	43385	GH	B.1.429
SARS-CoV-2	43386	G	B.1.525
SARS-CoV-2	43387	GH	B.1.526
SARS-CoV-2	43388	GR	P.1
SARS-CoV-2	43389	G	B.1.617.1
SARS-CoV-2	43404	G	B.1.620
SARS-CoV-2	43405	GK	B.1.617.2
SARS-CoV-2	43407	GH	B.1.621
SARS-CoV-2	43408	GRA	BA.1
SARS-CoV-2	43411	GRA	BA.1.1
SARS-CoV-2	43412	GRA	BA.2
SARS-CoV-2	43423	GRA	BA.2.12.1
SARS-CoV-2	43424	GRA	BA.2.3
SARS-CoV-2	43425	GRA	BA.4
SARS-CoV-2	43426	GRA	BA.5

* GSAID (Global Initiative on Sharing All Influenza Data)

Table S2. The SARS-CoV-2 RT-LAMP primers used in this study.

Primer set	Name	Sequence (5′-3′)	μM
Primer mix A	RdRP F3	CCG ATA AGT ATG TCC GCA AT	4
	RdRP B3	GCT TCA GAC ATA AAA ACA TTG T	4
	RdRP FIP	ATG CGT AAA ACT CAT TCA CAA AGT CCA ACA CAG ACT TTA TGA GTG TC	32
	RdRP BIP	TGA TAC TCT CTG ACG ATG CTG TTT AAA GTT CTT TAT GCT AGC CAC	32
	RdRP LF	TGT GTC AAC ATC TCT ATT TCT ATA G	10
	RdRP LB	TCA ATA GCA CTT ATG CAT CTC AAG G	4
	RdRP LB_FAM probe	[FAM]- CGG GCC CGT ACA AAG GGA ACA CCC ACA CTC CGT CAA TAG CAC TTA TGC ATC TCA AGG	6
	Quencher probe	GAG TGT GGG TGT TCC CTT TGT ACG GGC CCG-BHQ1	8
Primer mix B	RdRP F3	CCG ATA AGT ATG TCC GCA AT	4
	RdRP B3	GCT TCA GAC ATA AAA ACA TTG T	4
	RdRP FIP biotin	[Biotin]- ATG CGT AAA ACT CAT TCA CAA AGT CCA ACA CAG ACT TTA TGA GTG TC	32
	RdRP BIP biotin	[Biotin]- TGA TAC TCT CTG ACG ATG CTG TTT AAA GTT CTT TAT GCT AGC CAC	32
	RdRP LF	TGT GTC AAC ATC TCT ATT TCT ATA G	10
	RdRP LB_DIG probe	[Digoxigenin]- TCA ATA GCA CTT ATG CAT CTC AAG G	10

Table S3. Results of Allplex™ SARS-CoV-2 Assay, SARS CoV-2 RT-LAMP and Rapid SARS CoV-2 RT-LAMP-LFA kit for 1 wild and 19 mutations of SARS CoV-2 test.

SARS CoV-2 variants (10^5 PFU mL^{-1})	NCCP No.	Sensitivity				
		Allplex™ SARS-CoV-2 Assay		SARS CoV-2 RT-LAMP		Rapid SARS CoV-2 RT-LAMP-LFA kit
		Ct[1]	RFU[2]	Ct	RFU	P/N[3]
Wild type	43346	14.08	7077	6.19	4851	P
B.1.1.7	43381	15.43	11700	6.41	5070	P
B.1.351	43382	16.39	11216	7.01	4818	P
P.2	43383	12.47	12571	6.03	4861	P
B.1.427	43384	16.53	12450	7.14	4949	P
B.1.429	43385	15.98	12807	7.21	4823	P
B.1.525	43386	22.56	12532	9.06	5392	P
B.1.526	43387	21.21	11736	8.67	4065	P
P.1	43388	13.84	11577	6.39	4679	P
B.1.617.1	43389	17.70	5337	7.15	4478	P
B.1.620	43404	16.52	6717	6.48	4313	P
B.1.617.2	43405	19.46	2672	7.51	4699	P
B.1.621	43407	20.25	5817	7.63	5040	P
BA.1	43408	14.09	3608	5.77	5434	P
BA.1.1	43411	18.59	3477	7.39	4742	P
BA.2	43412	18.52	3319	7.66	4422	P
BA.2.12.1	43423	17.91	3386	7.23	4720	P
BA.2.3	43424	21.65	3717	8.60	4636	P
BA.4	43425	20.84	3979	8.25	4481	P
BA.5	43426	23.36	3314	9.22	4279	P

[1] Ct; Cycle threshold, [2] RFU; Relative fluorescence unit, [3] "P" and "N" indicate positive and negative of the reaction, respectively.

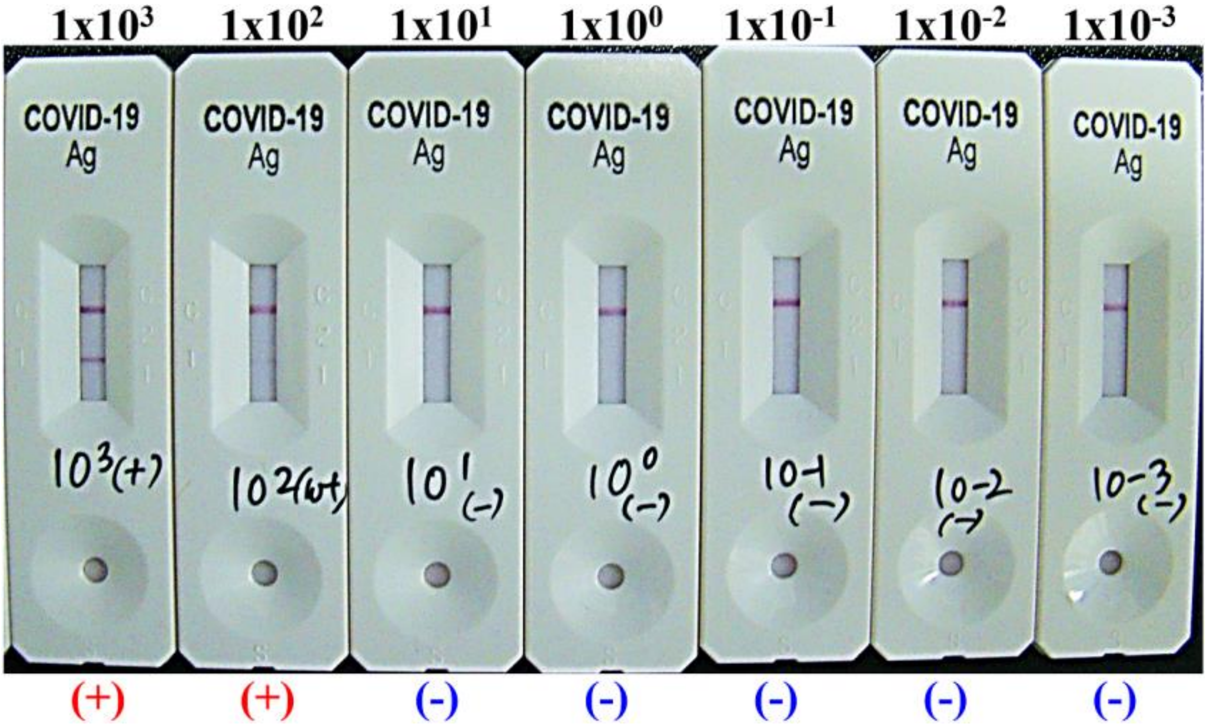

Figure S1. Limit of detection test of the rapid antigen kit for SARS CoV-2 (NCCP 43346, wild-type) spiked in normal clinical nasopharyngeal samples (range of 10^{3}-10^{-3} PFU ml^{-1}). "+" and "-" indicate positive and negative of the reaction, respectively.

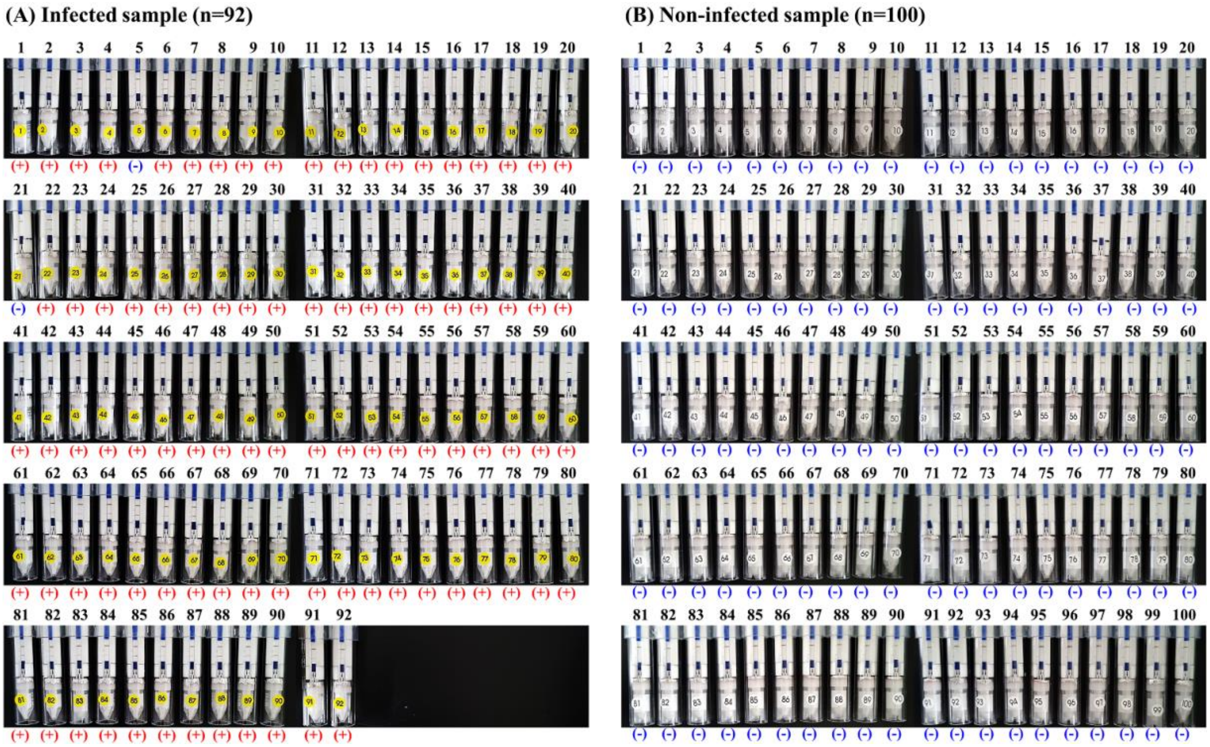

Figure S2. Results of clinical performance of the rapid SARS CoV-2 RT-LAMP-LFA kit for clinical samples. (A) 92 NP swab samples from individuals with SARS CoV-2. (B) 100 clinical NP swab samples from individuals without viral respiratory infections. “+” and “-” indicate positive and negative of the reaction, respectively.

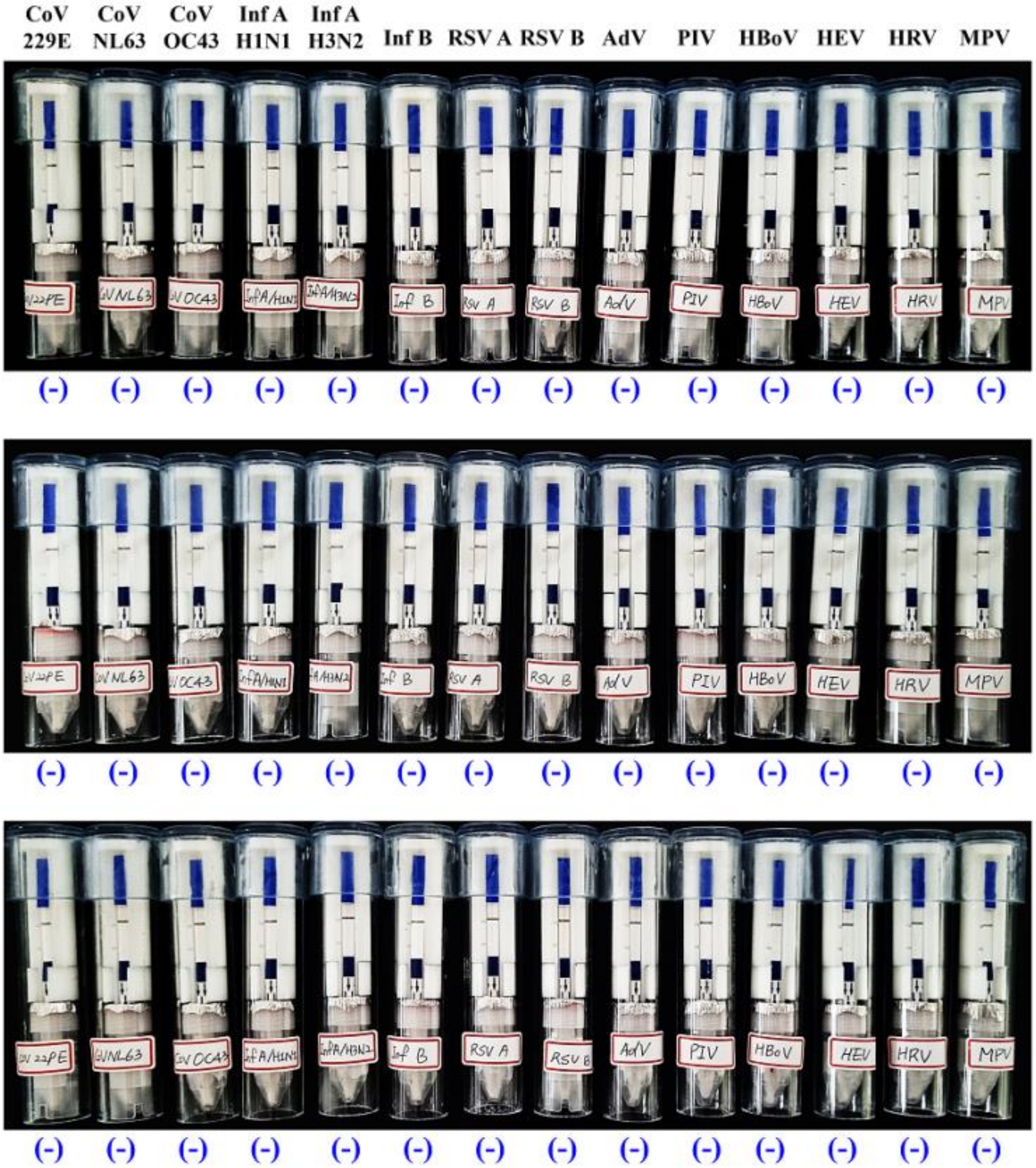

Figure S3. Results of Cross-reactivity of the rapid SARS CoV-2 RT-LAMP-LFA kit for SARS CoV-2 against other human infectious viruses. "+" and "-" indicate positive and negative of the reaction, respectively.

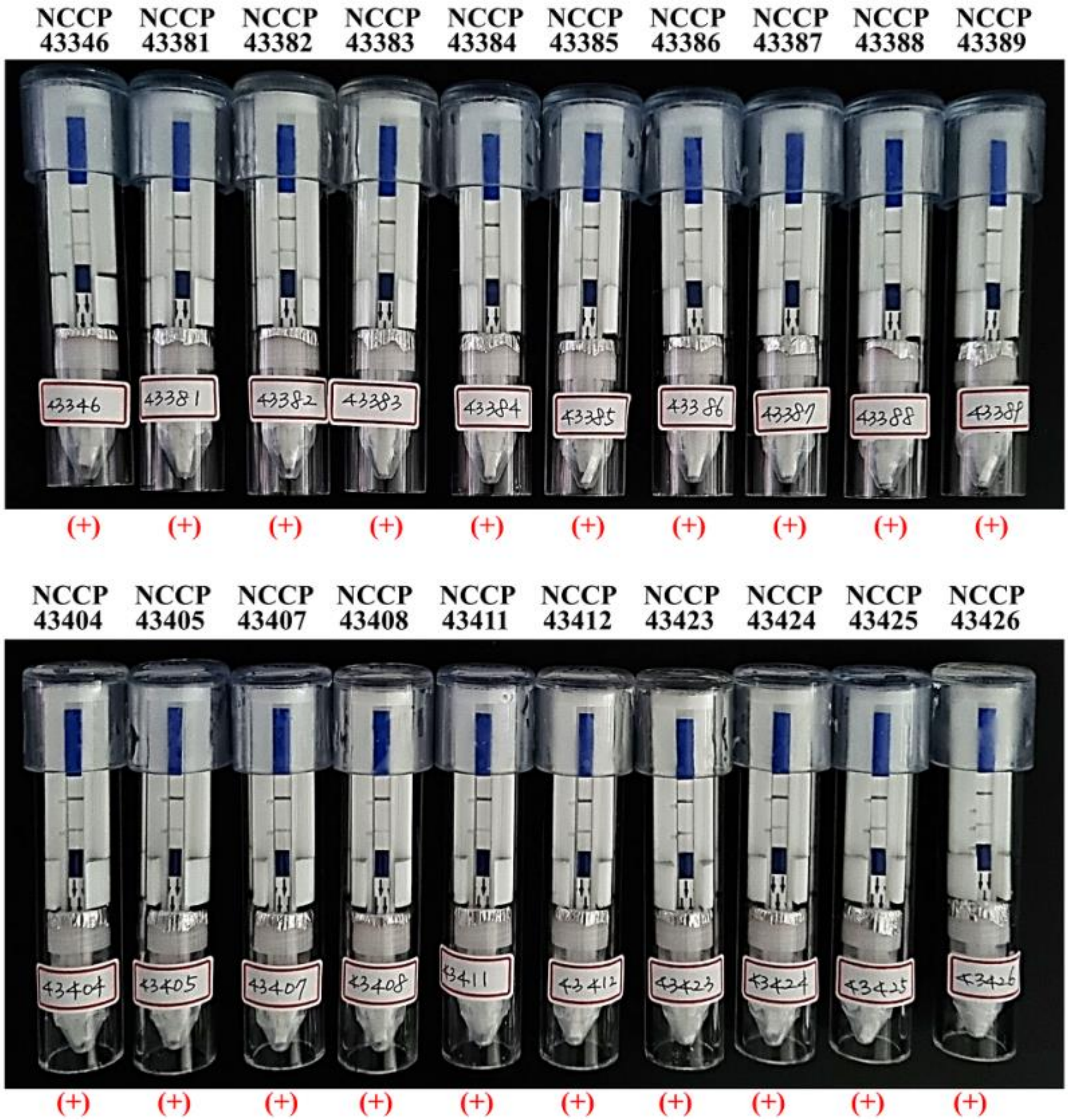

Figure S4. Results of the rapid SARS CoV-2 RT-LAMP-LFA kit for SARS CoV-2 for 1 wild and 19 mutations of SARS CoV-2 test. "+" and "-" indicate positive and negative of the reaction, respectively.

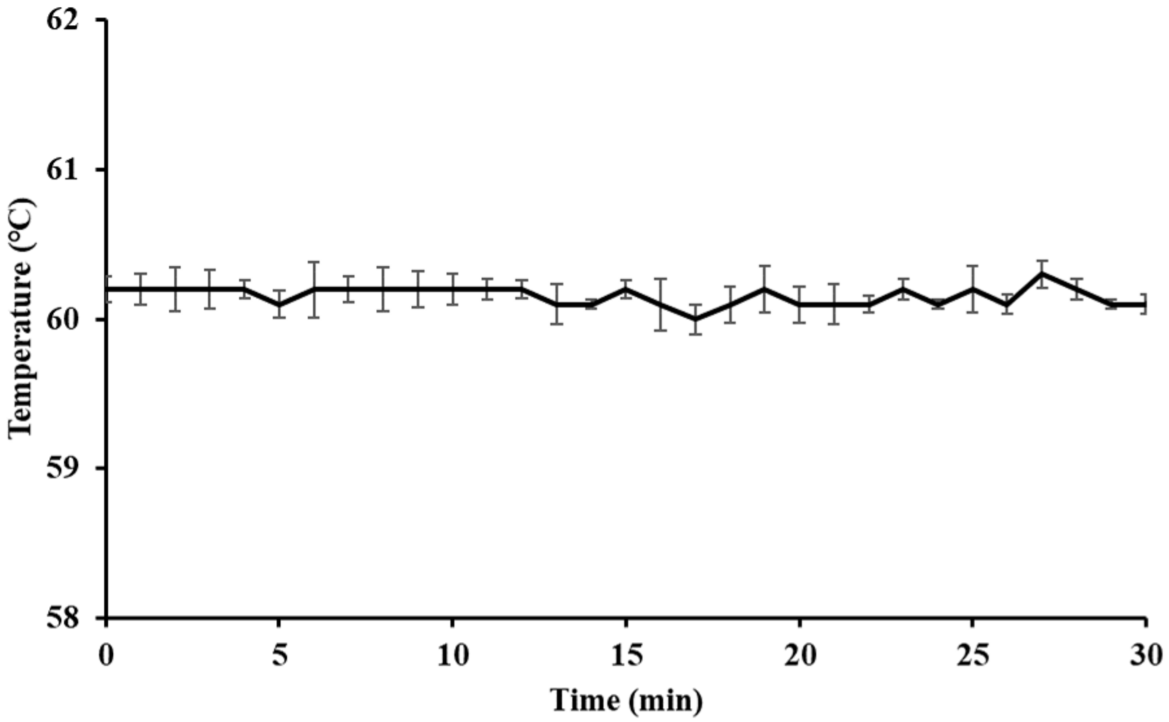

Figure S5. Temperature changes of the heat block. The tests were repeated 3 times.
